# Review of *Neopalpa* Povolný, 1998 with description of a new species from California and Baja California, Mexico (Lepidoptera, Gelechiidae)

**DOI:** 10.3897/zookeys.646.11411

**Published:** 2017-01-17

**Authors:** Vazrick Nazari

**Affiliations:** 1 3058-C KW Neatby Building, 960 Carling Avenue, Ottawa, ON K1A 0C6 Canada

**Keywords:** Microlepidoptera, new species, nomenclature, taxonomy, Donald J. Trump

## Abstract

The monotypic genus *Neopalpa* was described in 1998 by Czech entomologist Dalibor Povolný based on two male specimens from Santa Catalina Island, California, which he named *Neopalpa neonata*. The female of this species was discovered recently based on a DNA barcode match and is described. In addition, a new species with marked differences in morphology and DNA barcodes from southern California and Baja California Mexico is described as *Neopalpa donaldtrumpi* **sp. n.** Adults and genitalia of both species are illustrated, new diagnosis for the genus *Neopalpa* is provided, and its position within Gelechiidae is briefly discussed.

## Introduction

The tribe Gnorimoschemini currently consists of 44 genera, only six of which have exclusively Nearctic distributions (Povolný 2002). The defining characters of Gnorimoschemini remain vague since it was defined based on genital morphology with no synapomorphies proposed (Povolný 1964, Huemer and Karsholt 2010), although the reduction of muscle m7 in male genitalia maybe a unifying trait (Kuznetsov and Stekolnikov 2001, Povolný 2002). A combination of characters have been suggested that weakly support the monophyly of Gnorimoschemini, namely the presence of a hook-like signum and a ventromedial zone of microtrichia near the ostial area of the female genitalia (Huemer and Karsholt 1999, Ponomarenko 2006); however, one or both of these traits are sometimes absent in some genera (e.g., *Symmetrischema*). The monophyly and higher classification of the tribe thus remains unresolved.

In November 2011, a non-descript female gelechiid moth collected by J.-F. Landry from Santa Cruz Island in 1984 (CNCLEP00077350) was submitted for DNA barcoding. This specimen yielded a unique 407 bp fragment COI barcode that, while clustering with other Gnorimoschemini specimens in Neighbour-Joining analyses, morphologically did not match any of the known species of Gnorimoschemini that I had studied. In May 2012 additional matching barcode sequences from a Malaise trap catch in California became available that contained samples from both sexes. The genitalia of the females from this series were identical to the CNC specimen, and dissection of the male revealed its identity as *Neopalpa neonata* as described and illustrated by Povolný in 1998. Subsequently I examined the holotype and the only paratype of this species and confirmed the identification. Many additional specimens were later collected and barcoded from California and Arizona, mainly through the continuous Malaise trapping initiative by the Biodiversity Institute of Ontario BioBus program.

The new species was initially discovered through dissection of Gnorimoschemini material borrowed from the Bohart Museum of Entomology, University of California, Davis (UCBME). Two males and one female from Algodones dunes in Imperial County, California, showed a unique genitalia and wing pattern that did not match known species of Gnorimoschemini. Based on similarities in genitalia of both sexes I associated these with *Neopalpa*, and this identification was further supported by DNA barcoding. A few additional male specimens were later discovered among the material borrowed from other institutions. In this paper I provide a new diagnosis for the genus *Neopalpa*, illustrate the previously unknown female of *Neopalpa neonata* Povolný, 1998, and describe this newly discovered species.

## Materials and methods

***Collections*.** Specimens were examined from the following collections:

BIOUG
 Biodiversity Institute of Ontario, University of Guelph, Ontario, Canada 


CNC
 Canadian National Collection of Insects, Ottawa, Ontario, Canada 


Albu
 Research collection of Valeriu Albu, O’Neals, California, USA 


CAS
 California Academy of Sciences, San Francisco, USA 


EMEC
 Essig Museum of Entomology, University of California, Berkeley, USA 


LACM
 Natural History Museum of Los Angeles County, Los Angeles, California, USA 


UCR
 Entomology Research Museum, University of California, Riverside, California, USA 


UCBME
 Bohart Museum of Entomology, University of California, Davis, California, USA 


***Dissections*.** Genitalia dissections and slide mounts followed procedures outlined in Landry (2007). Intact male genitalia were excised and photographed in lactic acid after cleaning and removal of scales. The unrolling technique (Pitkin 1986, Huemer 1988) was used for male slide preparations. The male genitalia slide of the holotype was dissolved, and genitalia also unrolled and remounted. Intact (in lactic acid) and slide-mounted genitalia were photographed with a Nikon DS-Fi1 digital camera mounted on a Nikon Eclipse 800 microscope at magnifications of 100×. Nikon’s NIS 2.3 Elements was used to assemble multiple photos of different focal planes into single deep-focus images. The descriptive terminology of genitalia structures generally follows Huemer and Karsholt (2010) and Kristensen (2003).

***Photography*.** Pinned specimens were photographed with a Canon EOS 60D with a MP-E 65 mm macro lens. They were placed on the tip of a thin plastazote wedge mounted on an insect pin, with the head facing toward the pin and the fringed parts of the wings facing outward. This ensured that there was nothing between the fringes and the background. All specimens were photographed over a white background. Lighting was provided by a ring of 80 LED covered with a white diffuser dome (Fisher 2012 and references therein). The camera was attached to a re-purposed stereoscope fine-focusing rail. Sets of 20–35 images in thin focal planes were taken for each specimen and assembled into deep-focused images using Zerene Stacker and edited in Adobe Photoshop.

***DNA sequence analysis*.** DNA extracts were prepared from one or two legs removed from each specimen. DNA extraction, PCR amplification of the barcode region of COI, and subsequent sequencing followed standard protocols at the Canadian Centre for DNA Barcoding (deWaard et al. 2008). Attempts to barcode the type specimens of *Neopalpa neonata* failed due to their old age. All resultant sequences, along with the voucher data, images, and trace files, are publicly available in the BOLD dataset “DS-NEOPALPA”, https://doi.org/10.5883/DS-NEOPALPA. Sequences > 600 bp are deposited in GenBank (for accessions, see Suppl. material 1). Uncorrected p-distances were calculated in MEGA5 (Tamura et al. 2011). To infer a phylogenetic framework for Gnorimoschemini, a nexus file of 35 sequences, including several outgroups and one representative from 32 Gnorimoschemini genera, together with the sequences from the two species of *Neopalpa*, was compiled using public sequences on BOLD and subjected to a Bayesian analysis of 50m generations in MrBayes v.3.2. (Ronquist et al. 2012) under the *GTR + Γ + I* model with two simulations, independent Markov chain Monte Carlo (MCMC) runs starting from different random trees, each with three heated chains and one cold chain.

## Results

### Neopalpa

Taxon classificationAnimaliaLepidopteraGelechiidae

Povolný, 1998

Neopalpa Povolný, 1998a: 141, figs 1, 6.

#### Type species.

*Neopalpa neonata* Povolný, 1998b (by original designation).

#### Diagnosis.

*Neopalpa* can be defined by the combination of the following genitalia characters: male tegumen long and parallel sided, gnathos a short and delicate spine with distinct V-shaped arms, uncus tall and rounded, valvae sigmoid with antler-shaped tip, saccus narrow and nearly as long as tegumen, phallus nearly as long as longitudinal axis of genitalia, with a subovate caecum and a sub-terminal projection. Female with a well-developed antrum and an aviform signum.

#### Remarks.

Povolný (1998a) did not provide a differential diagnosis for *Neopalpa neonata*; instead he emphasized the “extremely characteristic” male genitalia, “… mainly reflected in the form of the curious bilobate paired process arising from the sacculus, in the shape of slender parallel-sided sigmoid valva with its curious tip, and in the long slender aedeagus with the specialized tip.” As for the adult, he commented, “The moth shows the monotonous forewing coloration with indistinct blackish stigmata characteristic of the tribe.” With the discovery of a new species of *Neopalpa* described here, none of these characters holds up as a synapomorphy for the genus. While comparable structures occur in other Gnorimoschemini (e.g., some species in *Ephysteris* or *Keiferia*), the “curious bilobate paired process arising from the sacculus” is significantly reduced in the new species. Also, the sigmoid bifurcating valve is not unique in Gnorimoschemini (present in some species of *Keiferia*, *Scrobipalpopsis* and *Symmetrischema*), and the new species does not show a highly-specialized phallus. In addition, the adults of the new species show a highly-contrasting wing pattern that is very distinct from *Neopalpa neonata*.

#### Description.

*Head*. Scaled with light-yellow frons, scales on the vertex converging towards middle, often with darker tips, ocelli present, small, located behind the base of antenna. Labial palpi strongly up-curved, segment 3 acute, about 2/3 length of segment 2; antenna with more or less distinct dark and light rings.

*Thorax*. Grey to brown; wingspan 7–12 mm; forewing slender, discal and apical areas dark brown, termen with black-tipped white scales. Hindwing off-white to greyish with a well-developed tornal lobe and a pointed tip.

*Abdomen*. Male tergum 8 subtriangular, equilateral, weakly sclerotized and concave anteriorly; sternum 8 more than twice the size of tergum 8, subrectangular, broader than long, posterior margin broadly rounded, anterior margin bilobate with a deep ventral emargination. Female segment 7 trapezoidal, tergum 7 approximately twice the length of other abdominal segments; apodemes in both sexes well developed. Coremata absent.

*Male genitalia*. Characterized by elongate shape, long parallel-sided tegumen and slender, well-rounded uncus; gnathos a short spine; culcitula weakly developed; valva sigmoid, parallel sided with a bifurcating antler-shaped tip; sacculus parabasally located, short and cone shaped; vincular processes variously developed; saccus elongate, nearly as long as tegumen; phallus elongate with a subovate caecum and a distinct subterminal spine.

*Female genitalia*. Segment 8 with almost evenly sclerotized subgenital plate, moderately to strongly sculpted, ventromedial zone membranous; ostium bursae distinctly edged; antrum wide, tubular and weakly sclerotized, nearly ¾ length of apophysis anterioris; apophysis anterioris thin and parallel sided, about same length as segment 8; ductus bursae same width as antrum and same length as apophysis anterioris; corpus bursae clearly delineated, bulbous; signum aviform with a central spine (hook) and two wide subtriangular lobes.

#### Distribution.

Western USA (California and Arizona) and Baja California, Mexico.

#### Biology.

Both species specialize in xeric habitats. The host plant is unknown, but is probably in the Solanaceae (one specimen of *Neopalpa neonata* “collected in tomato foliage”).

#### Key to species of *Neopalpa*

**Table:** 

1	Forewing predominantly dark brown or gray; male genitalia with large bilobate vincular processes 4× length of sacculus; phallus with a curved tip and a distinct subterminal hook; female genitalia segment 8 extensively sculpted with microtrichea, signum aviform with granulated wings	***Neopalpa neonata***
–	Forewing orange yellow except costal and terminal areas dark brown; male genitalia vincular processes not longer than sacculus, phallus tip acute with a subtle subterminal thorn; female genitalia segment 8 with hardly any microtrichea, signum aviform with smooth wings	***Neopalpa donaldtrumpi* sp. n.**

### Review of species

#### Neopalpa neonata

Taxon classificationAnimaliaLepidopteraGelechiidae

Povolný, 1998

BOLD:ABW8320

**Figure 1.**
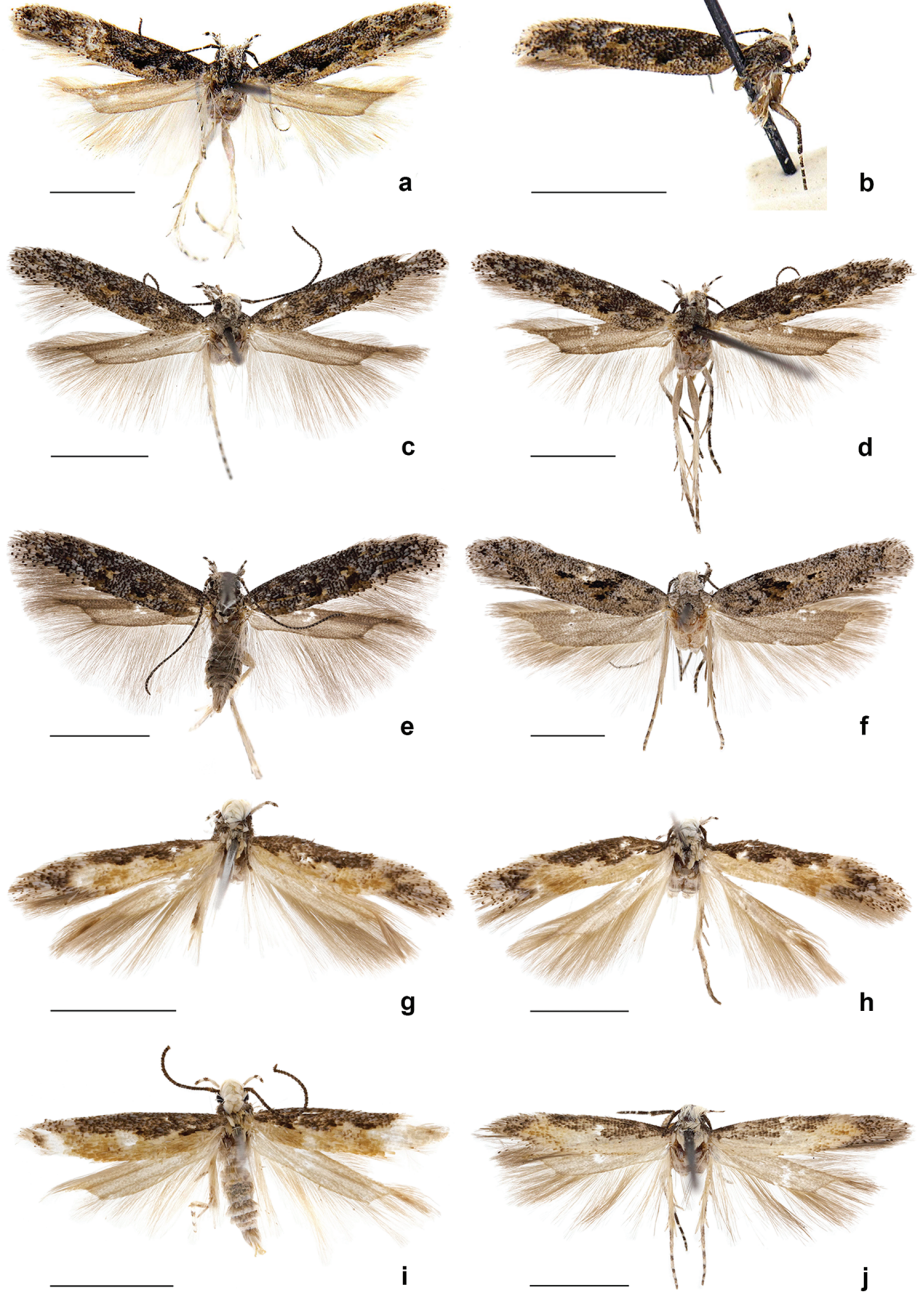
Adults of *Neopalpa* species. **a–f** *Neopalpa neonata* **g–j** *Neopalpa donaldtrumpi* sp. n. *Neopalpa neonata*: **a** holotype ♂ EMEC82306 (CA: Santa Catalina Island) **b** paratype ♂ EMEC342305 (Mexico: Baja California Sur) **c** ♀ CNCLEP00077350 (CA: Santa Cruz Island) **d** ♀ EMEC407544 (CA: Santa Cruz Island) **e** ♂ LACMENT326744 (CA: San Bernardino County) **f** ♀ EMEC408849 (CA: Modoc County); *Neopalpa donaldtrumpi* sp. n.: **g** Holotype ♂ UCBMEP0201628 (CA: Imperial County) **h** Paratype ♀ UCBMEP0201482 (CA: Imperial County) **i** Paratype ♂ UCBMEP0201629 (CA: Imperial County) **j** Paratype ♂ EMEC408498 (Mexico: Baja California Sur). For detailed specimen data see Suppl. material 1. Scale bar 2 mm.

**Figure 2.**
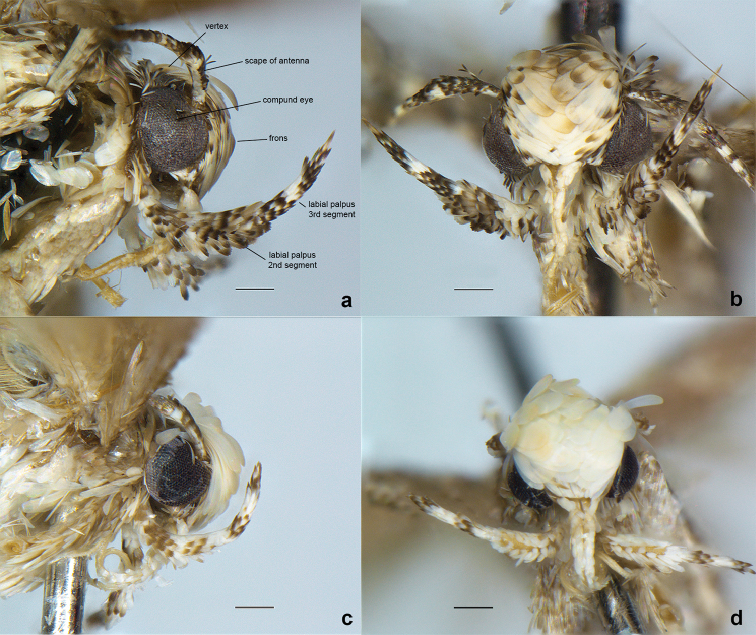
Close up of the head of male *Neopalpa* species. **a, b** *Neopalpa neonata* (LACMENT326885, Mexico: Baja California) **c, d** *Neopalpa donaldtrumpi* sp. n., holotype (UCBMEP0201628, CA: Imperial County). Left: lateral aspect, right: frontal aspect. Scale bar 1 mm.

**Figure 3.**
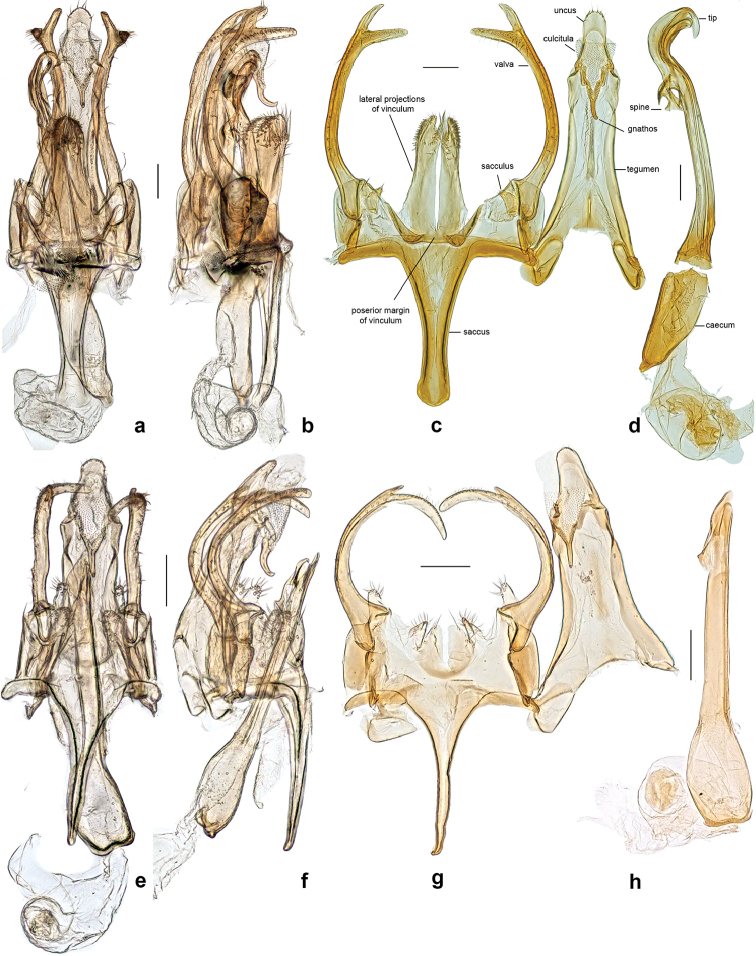
Male genitalia of *Neopalpa* species. **a–d** *Neopalpa neonata* **e–h** *Neopalpa donaldtrumpi* sp. n. **a, b** ventral and lateral view of intact genitalia, unmounted, stored in lactic acid (LACMENT326710, CA: Riverside County) **c, d** Unrolled genitalia and phallus of the Holotype (EMEC82306, dissection Pw1168) **e, f** ventral and lateral view of intact genitalia, unmounted, stored in lactic acid (UCRC ENT 461717, CA: Riverside County) **g, h** Unrolled genitalia and phallus of the Holotype (UCBME021628; dissection VNZ241). Scale bar 100 μm.

**Figure 4.**
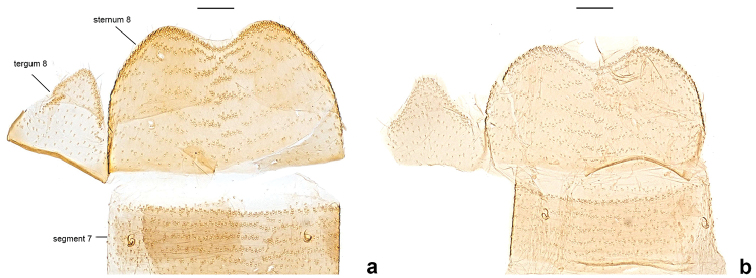
Last abdominal segment of male *Neopalpa* species. **a** *Neopalpa neonata* (specimen BIOUG01860-D04, dissection MIC6545; CA: San Diego County) **b** *Neopalpa donaldtrumpi* sp. n. (UCIS 313268, dissection VNZ556; CA: Riverside County). Scale bar 100 μm.

**Figure 5.**
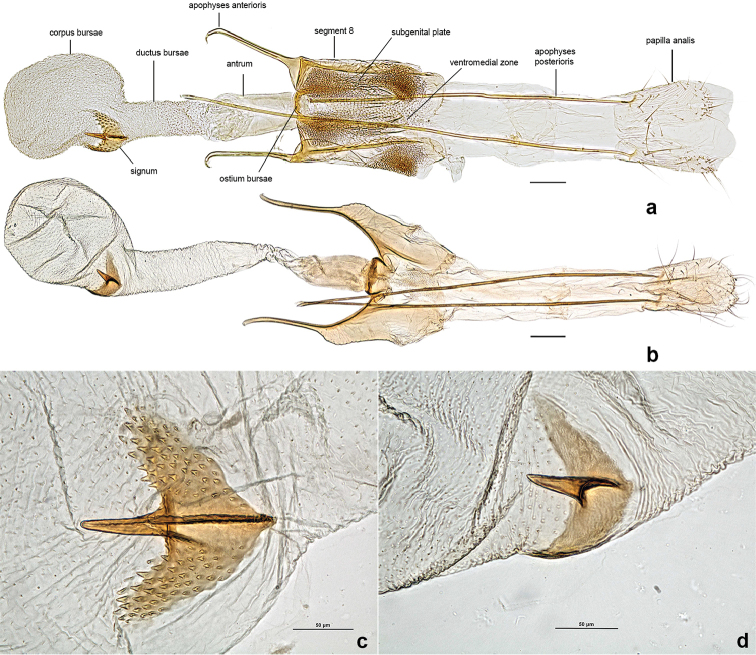
Female genitalia of *Neopalpa* species. **a** *Neopalpa neonata* (CNCLEP00077350, dissection MIC6530; CA: Santa Cruz Island) **b** *Neopalpa donaldtrumpi* sp. n. (UCBMEP0201482, dissection VNZ241; CA: Imperial County); Close-up of signa **c** *Neopalpa neonata* (UCBMEP0201495, dissection VNZ239; CA: Imperial County) **d** *Neopalpa donaldtrumpi* sp. n. (same as b). Scale bar 100 μm.

Figs 1a–f , 2a, b , 3a–d , 4a, c , 5a

Neopalpa neonata Povolný, 1998a: 141, figs 1, 6.

##### Material examined.

Holotype ♂, California: Los Angeles County, Santa Catalina Island, Middle Cyn. 5.ii.1978, J.A. Chemsak, specimen # EMEC82306, genitalia slide Pw1173 (EMEC). Paratype ♂, Mexico: Baja California Sur, 21 mi. W. La Paz, 8.9.1966, J.A. Powell, specimen # EMEC342305, genitalia slide Pw1173 (EMEC).

For a complete list of additional specimens examined from California, Arizona and Mexico (n = 386), see Suppl. material 1.

##### Diagnosis.

Distinguishable from the species described below by dark forewing and frons, well-developed vincular processes that are more than 4× as long as sacculus, segment 8 in female genitalia heavily sculpted with microtrichea, and signum heavily granulated with small stubby spines.

##### Redescription.

***Adult*** (Figs 1a–f, 2a, b). Forewing length: male 3.6–4.9 mm (mean 4.2 mm, n=50); female 4.2–5.0 mm (mean 4.3 mm, n=50). Head, thorax and tegula covered with a mixture of grayish-brown scales with darker tips; scales on vertex and frons with darker tips, often appressed, converging towards middle. Labial palpi up-curved, annulated, segment 3 acute, about ⅔ size of segment 2; antenna about ⅔ length of forewing, with more or less distinct dark and light rings, scape covered with yellow and dark-brown scales. Mesoscutum grayish brown, tegulae greyish brown to brown. Forewing upper surface ground coloration consists of a mixture of grayish-brown scales with dark tips, the dorsal region and subterminal fascia distinctly paler partly mixed with orange scales; three to four obscure black tear-shaped stigmata situated axially, first near forewing base, second in wing center, third in cell; additional one or two stigmata in the dorsal forewing fold, the first near wing center, the second rather indicated by a group of chocolate-brown scales before external transverse band near forewing dorsum; apical area and fringes generally dark grey mottled with lighter suffusion. Hindwing deep grey, unmarked, slender, with distinctly protruding tip. Sexes similar.

***Variation*.** Adult size and the intensity of forewing pattern variable. A large female specimen (wingspan=6.3mm, not included in mean wingspan calculation) from Cedar Pass Campground, Modoc County (EMEC408849, dissection VNZ 591, Fig. 1f) shows gray ground coloration on wings instead of dark brown, but the female genitalia are identical to those of *Neopalpa neopalpa* (Fig. 1f).

***Male genitalia*** (Figs 3a–d, 4a) (seven preparations examined). Tergum 8 subtriangular, equilateral, weakly sclerotized and concave anteriorly; sternum 8 more than 2× the width of tergum 8, subquadrate, broader than long, posterior margin broadly rounded, anterior margin bilobate with a protruded anterolateral corner.

Tegumen slender, parallel-sided, the anterior margin laterally notched; uncus long and narrow with a round apex, finely setose; gnathos a short delicate spine with distinct V-shaped arms about same width; culcitula weakly developed. Valva sigmoid, long and slender, parallel-sided, bifurcating at about ⅘ length of valva towards tip into two equally-sized acute branches in form of antlers; sacculus parabasally located, short and cone-shaped, finely setose; posterior margin of vinculum with a striking paired bilobate process separated by a deep, narrow excision; this process with a shorter truncate lobe provided with a brush of long, stiff hairs, their tips extending over second (longer) lobe with obtuse upper edge and with rounded lateral side, both armoured with numerous short spinules; lateral side of the paired process fused basally with oblique wall of sacculus; (ventral) wall of sacculus strengthened basally by a distinct, paired, heavily-sclerotized curved ledge; saccus elongate, ligulate, nearly as long as tegumen, with distinctly-sclerotized lateral edge and a moderately rounded tip; phallus nearly as long as the longitudinal genitalia axis, moderately sigmoid, with a strongly-curved, rounded tip, a distinct subterminal hook-like spine, and an irregularly subovate caecum.

***Female genitalia*** (Fig. 5a, c) (eight preparations examined). Segment 8 parallel sided on slide mounts, subgenital plate heavily sculpted with microtraechia, two distinct dense round patches of microtraechia on Segment 8 posterolaterally; ostium bursae subspherical, distinctly-edged anterolaterally; antrum wide, tubular, weakly sclerotized, nearly ¾ length of apophysis anterioris; apophysis anterioris thin, parallel sided, about the same length as segment 8; ductus bursae about same width and length of antrum, lightly sculpted with speckles; corpus bursae clearly deliniated, bulbous, lightly sculpted; signum aviform with a central spine and two subtriangular wings covered with several rows of spinules.

##### Distribution.

Povolný’s assumption that this species is a Channel Islands endemic is incorrect, as it seems to have a much wider distribution on the mainland. Examined specimens are from the USA: California (Imperial, Inyo, Los Angeles, Modoc, Riverside, San Bernardino, San Diego, Santa Barbara and Solano counties); Arizona (Cochise and Coconino counties), as well as Mexico (States of Sonora, Baja California, and Baja California Sur).

##### Biology.

Adults fly throughout the year, probably in more than one generation. Studied specimens were collected in every month of the year in almost even numbers (the high number of samples collected in March is likely an artefact of high-volume Malaise trapping in one location). The two males that formed the type series of this species were collected in the “xeric maritime habitats extending from California Channel Islands.” Additional specimens examined were collected in a variety of generally dry habitat types in canyons, creeks, campgrounds, microphyll forest, dry bush, dunes and desert habitats. The life history and host plant remain unknown; nearly all specimens examined were collected at light or in Malaise traps, although one specimen (UCREM 4318, UCR) was “collected in tomato foliage.”

#### Neopalpa donaldtrumpi

Taxon classificationAnimaliaLepidopteraGelechiidae

Nazari sp. n.

http://zoobank.org/5FA78DB3-9FB8-409A-AD01-34567BB3C396

BOLD:ACR1768

Figs 1g–j , 2c–d , 3e–h , 4b,d , 5b

##### Type material.

***Holotype*** ♂: [label 1] “USA: CA: Imperial Co. | Algodones Dunes – Niland- | Glamis Rd. 7.4 km NW Glamis”, [label 2] “33°02N 115°08.3W | 21-25 April 2009 AL173 | Bohart Museum Team”, [label 3] “UC BME | P 0201628”, [label 4] “Barcode of Life Project | Leg(s) removed | DNA extracted”, [label 5] “genitalia slide | VNZ240 ♂.” Condition of specimen: double mounted, wings partly spread, left antenna and part of right antenna missing, left hind- and all right legs missing, partly removed for DNA barcoding. Deposited at UCBME.

***Paratypes*.** 5 males, 1 female. 1 ♂ same data as for holotype, specimen # UCBMEP0201629 (CNC); 1 ♀ **USA**: CA: Imperial Co., Algodones Dunes, Mammoth Wash, Niland-Glamis Rd., 29 km N. Hwy 78, 2008AL20, 6-9.II.2008, Malaise trap, S.L. Heydon & T.J. Zavortnik, specimen # UCBMEP0201482, slide VNZ241 (UCBME); 1 ♂ CA: Riverside Co., P.L. Boyd Desert Research Center, 3.5 miles S. Palm Desert, 13–18.VI.1969, Malaise trap, Saul Frommer & R. Worley, specimen # UCREM 18373, slide VNZ580 (UCR); 1 ♂ *ibid*, 16–17.8.1970, P.L. Boyd, specimen # UCRC ENT 461717 (UCR); 1 ♂ CA: Imperial Co., Deep Canyon, Coyote Creek, 5.IV.1975, J.B. Tucker, specimen # UCIS 313268, slide VNZ556 (UCR). 1 ♂ **Mexico**: Baja California Norte, Arroya Catavina, Hwy 1, 35 mi S Progresso, 1.IV.1976, Blacklight, P.A. Rude, specimen # EMEC408498, slide VNZ327 (EMEC).

##### Diagnosis.

The new species can be easily distinguished from *Neopalpa neonata* by its external appearance, the yellowish-white scales covering the frons of the adult head, and the distinctive orange-yellow coloration on the forewing dorsum. In the male genitalia, the valvae are strongly curved, the saccus has an acute tip, and the highly-developed bilobed processes of the vinculum, characteristic of *Neopalpa neonata*, are absent. In the female genitalia, the subgenital plate is simpler than in *Neopalpa neonata* and much less sculptured with microtrichea, and the signum wings are smooth.

##### Description.

***Adult*** (Figs 1g–j, 2c, d). Forewing length: male 3.0–4.6 mm (mean 3.6 mm, n=6); female 4.3 mm (n=1). Head and thorax off-white, tegula greyish brown to brown, scales on vertex and frons yellowish white, often rough, converging towards middle. Labial palpi strongly up curved, annulated, segment 3 slender and acute, about ⅔ size of segment 2; antenna about ⅔ length of forewing, with more or less distinct dark and light rings, scape covered with yellow and light-brown scales. Forewing upper surface with costal region dark brown with sparse, lighter speckles; dorsal region and discal fascia orange yellow to pale buff, the sinuous margin with two or three scallops; apical area and fringes dark brown heavily mottled with lighter suffusion. Hindwing pale buff, unmarked, with slightly darker fringe. Sexes similar.

***Male genitalia*** (Figs 3e–h, 4b) (four preparations examined). Tergum 8 subpentagonal, weakly sclerotized and concave anteriorly; sternum 8 more than 2× the width of tergum 8, subquadrate, broader than long, posterior margin broadly rounded, anterior margin bilobate with a protruded anterolateral corner. Genitalia comparatively smaller than for *Neopalpa neonata*, tegumen slender and parallel sided, anterior margin laterally notched, uncus long and narrow with a round tip; gnathos a short spine with distinct V-shaped arms about same width; culcitula weakly developed. Valva sigmoid, parallel sided, with a short spine at about ⅔ length towards tip; sacculus parabasally located, short and cone shaped; vinculum with lateral projections spine shaped and about the same size as sacculus, vinculum posterior margin weakly developed with a shallow anteromedial incision; saccus elongate, nearly as long as tegumen, narrowing towards an acute tip; phallus elongate with a subovate caecum and a distinct subterminal spine.

***Female genitalia*** (Fig. 5b, d) (1 preparations examined). Segment 8 with almost evenly sclerotized subgenital plate, with ventromedial zone membranous and moderately sculpted with microtraechia; ostium bursae subtriangular, distinctly edged anterolaterally; antrum wide, tubular and weakly sclerotized, nearly ¾ length of apophysis anterioris; apophysis anterioris thin and parallel sided, about same length as segment 8; ductus bursae about same width and 2× length of antrum, lightly sculpted with wrinkles; corpus bursae clearly deliniated, bulbous, lightly sculpted; signum aviform with a central spine and two smooth subtriangular wings.

##### Etymology.

The new species is named in honor of Donald J. Trump, to be installed as the 45^th^ President of the United States on January 20, 2017. The reason for this choice of name is to bring wider public attention to the need to continue protecting fragile habitats in the US that still contain many undescribed species. The specific epithet is selected because of the resemblance of the scales on the frons (head) of the moth to Mr. Trump’s hairstyle. The name is a noun in the genitive case.

##### Distribution.

So far only known from Riverside and Imperial counties in southern California and Baja California in Mexico.

##### Biology.

Specimens collected at mercury-vapour light, black-light or Malaise trap in February, April, June and August, in dry or sandy habitats. Life history and host plant unknown.

## Discussion

**Figure 6.**
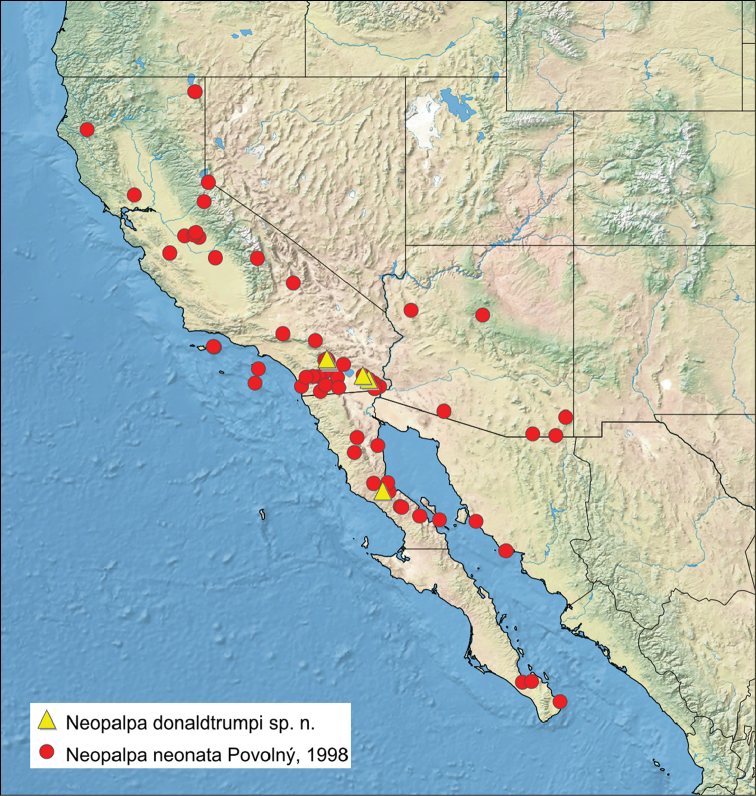
Distribution of *Neopalpa* species. (Created using simplemappr.net, accessed December 2016)

**Figure 7.**
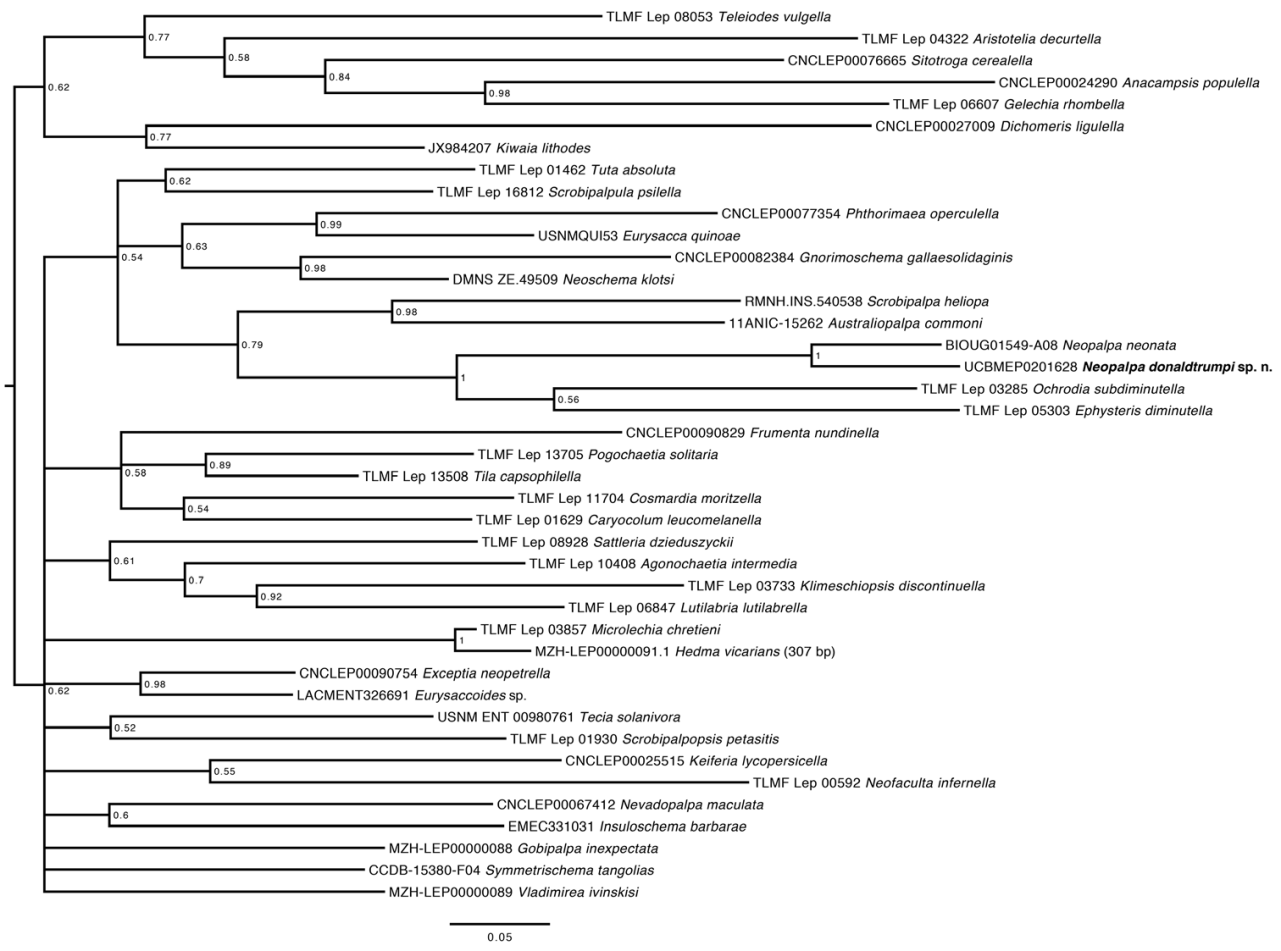
Bayesian Inference of COI barcode sequences for selected Gnorimoschemini species. Values are posterior probabilities for each node.

The two species of *Neopalpa* fly sympatrically in three localities in southern California (Deep Canyon and P.L. Boyd Desert Research Center in Riverside County, and Algodones dunes in Imperial County) (Fig. 6), although none of the examined specimens of *Neopalpa donaldtrumpi* were collected synchronically with *Neopalpa neonata* (see Suppl. material 1). The DNA barcodes of *Neopalpa neonata* and *Neopalpa donaldtrumpi* are 4.9–5.1% divergent from one another and are placed in separate BINs on BOLD. A Bayesian analysis of DNA barcodes from representatives of all available Gnorimoschemini genera placed these two species together, and as a distant sister to *Ochrodia* Povolný, 1966 and *Ephysteris* Meyrick, 1908 with good support. The tree was rooted using representative from other tribes of Gelechiinae, and other subfamilies of Gelechiidae. Short mitochondrial fragments such as the DNA barcode region cannot resolve deeper relationships at the subfamily level, as evident by these results. In the inferred phylogeny, all genera currently recognized under Gnorimoschemini (*sensu* Povolný 2002) group together in a large unresolved clade with weak support, except *Kiwaia* Philpott, 1930; in addition, *Neofaculta* Gozmany, 1955 (Gelechiinae: Chelariini) appears within the Gnorimoschemini clade as sister to *Keiferia* Povolný, 1967 (Fig. 7). *Ochrodia* has been treated as a valid genus (Povolný 2002, Huemer and Karsholt 2010) or a subgenus of *Ephysteris* (Karsholt and Sattler 1998, Li and Bidzylia 2008). These two taxa share the same “Ephysteroid” type of genitalia (*sensu* Povolný and Šustek 1988) with *Neopalpa*, and appear to be the most similar group to it within Gnorimoschemini.

The discovery of this distinct micro-moth in the densely populated and otherwise zoologically well-studied southern California underscores the importance of conservation of the fragile habitats that still contain undescribed and threatened species, and highlights the paucity of interest in species-level taxonomy of smaller faunal elements in North America. By naming this species after the 45^th^ President of the United States, I hope to bring some public attention to, and interest in, the importance of alpha-taxonomy in better understanding the neglected micro-fauna component of the North American biodiversity.

## Supplementary Material

XML Treatment for Neopalpa

XML Treatment for Neopalpa neonata

XML Treatment for Neopalpa donaldtrumpi

## Supplementary materials

Supplementary material 1 Material examined and GenBank accessions Data type: species data Explanation note: Material examined and GenBank accessions. This dataset is made available under the Open Database License (http://opendatacommons.org/licenses/odbl/1.0/). The Open Database License (ODbL) is a license agreement intended to allow users to freely share, modify, and use this Dataset while maintaining this same freedom for others, provided that the original source and author(s) are credited.Vazrick Nazari
